# Memory for Spatial Locations in a Patient with Near Space Neglect and Optic Ataxia: Involvement of the Occipitotemporal Stream

**DOI:** 10.3389/fneur.2017.00231

**Published:** 2017-05-31

**Authors:** Sergio Chieffi, Giovanni Messina, Antonietta Messina, Ines Villano, Vincenzo Monda, Ferdinando Ivano Ambra, Elisabetta Garofalo, Felice Romano, Maria Pina Mollica, Marcellino Monda, Alessandro Iavarone

**Affiliations:** ^1^Department of Experimental Medicine, University of Campania “Luigi Vanvitelli”, Naples, Italy; ^2^Department of Clinical and Experimental Medicine, University of Foggia, Foggia, Italy; ^3^Neurological and Stroke Unit, CTO Hospital, AORN “Ospedali dei Colli” Naples, Naples, Italy; ^4^Department of Biology, Università degli Studi di Napoli Federico II, Naples, Italy

**Keywords:** Balint syndrome, optic ataxia, neglect, spatial memory, attention, reaching, proprioception

## Abstract

Previous studies suggested that the occipitoparietal stream orients attention toward the near/lower space and is involved in immediate reaching, whereas the occipitotemporal stream orients attention toward the far/upper space and is involved in delayed reaching. In the present study, we investigated the role of the occipitotemporal stream in attention orienting and delayed reaching in a patient (GP) with bilateral damage to the occipitoparietal areas and optic ataxia. GP and healthy controls took part in three experiments. In the experiment 1, the participants bisected lines oriented along radial, vertical, and horizontal axes. GP bisected radial lines farther, and vertical lines more above, than the controls, consistent with an attentional bias toward the far/upper space and near/lower space neglect. The experiment 2 consisted of two tasks: (1) an immediate reaching task, in which GP reached target locations under visual control and (2) a delayed visual reaching task, in which GP and controls were asked to reach remembered target locations visually presented. We measured constant and variable distance and direction errors. In immediate reaching task, GP accurately reached target locations. In delayed reaching task, GP overshot remembered target locations, whereas the controls undershot them. Furthermore, variable errors were greater in GP than in the controls. In the experiment 3, GP and controls performed a delayed proprioceptive reaching task. Constant reaching errors did not differ between GP and the controls. However, variable direction errors were greater in GP than in the controls. We suggest that the occipitoparietal damage, and the relatively intact occipitotemporal region, produced in GP an attentional orienting bias toward the far/upper space (experiment 1). In turns, the attentional bias selectively shifted toward the far space remembered visual (experiment 2), but not proprioceptive (experiment 3), target locations. As a whole, these findings further support the hypothesis of an involvement of the occipitotemporal stream in delayed reaching. Furthermore, the observation that in both delayed reaching tasks the variable errors were greater in GP than in the controls suggested that in optic ataxia is present not only a visuo- but also a proprioceptivo-motor integration deficit.

## Introduction

Goodale and Milner (1–5) proposed the model of two cortical pathways of visual processing. According to their model, the dorsal (occipitoparietal) stream provides “vision for action” and the ventral (occipitotemporal) stream “vision for perception” (1, 3, 5). The two streams would process and transmit visual information in quite different ways, each stream contributing to elaborate selective components of action. The dorsal stream would compute object target location relative to some position on the body, such as the eye, head, trunk, shoulder, or hand (i.e., in egocentric coordinates), and provide for the subsequent implementation of the action. Its processing occurs in real time, i.e., with the target object visible during the programming phase (5). Conversely, when there is a delay between stimulus offset and the initiation of the reaching movement, the movement programming is driven by a memory of the target created by mechanisms in the ventral stream (5). In this case, the location of the object target is encoded relatively to the other objects in the visual environment, i.e., in allocentric coordinates (6, 7).

Clinical evidence shows that a lesion that damages selectively the dorsal or ventral pathway produces specific symptoms. A lesion of the ventral pathway may cause visual agnosia (8, 9). A patient with visual agnosia shows a gross impairment in recognition of visually presented objects. A lesion of the dorsal pathway may cause optic ataxia. A patient with optic ataxia shows a gross spatial inaccuracy in reaching toward individual objects in his visual field (10–12). In optic ataxia, patients with unilateral lesions show marked disorders when required to reach to an object presented in their contralesional/ataxic visual field (“field effect”), and also when using their contralesional/ataxic hand (“hand effect”). These reaching inaccuracies largely interact, that is, when the contralesional hand is used to reach within the contralesional visual field (13, 14), so amplifying the misreaching error. In general, reaching in central vision is spared (13, 14). Optic ataxia is considered to be a specific visuomanual guidance deficit. It would depend on an impairment of spatiomotor integration of visual targets locations (15). Interestingly, optic ataxic patients show a significant improvement in their pointing accuracy when a delay is interposed between the presentation of the stimulus and the signal to respond (16–18). It has been proposed that this improvement would occur because the patient now uses a memory of the stimulus location based on perceptual processing carried out at the time of stimulation by his relatively intact occipitotemporal cortex (16). A complementary dissociation was also reported. Milner et al. (19) described the case of DF, who presented bilateral damage of occipitotemporal cortex and visual-form agnosia. DF showed normal accuracy in an immediate pointing task, but her performance deteriorated in a delayed pointing task. Milner et al. (19) proposed that DF selectively failed to store, even for a few seconds, information about target location to-be-reached (19).

A further functional dissociation between the dorsal and ventral streams was reported in clinical setting. The study of single cases showed complimentary specializations of the ventral and dorsal pathways in orienting attention toward far/upper and near/lower spaces, respectively. These studies were performed by asking the participants to bisect lines (or rods) oriented radially or vertically. The patients with occipitoparietal lesions localized the center of radial and vertical lines, respectively, farther and more above than the healthy participants, suggesting the presence of neglect for near/lower space (20–22). Conversely, the patients with occipitotemporal lesions localized the subjective center of radial and vertical lines, respectively, nearer and more below than the healthy participants, suggesting the presence of neglect for far/upper space (23, 24). Drain and Reuter-Lorenz (25) hypothesized that the occipitoparietal and occipitotemporal streams are in mutually inhibitory control of attention orienting. The damage of the occipitoparietal regions would produce a far/upward attentional bias, being increased the activation of the occipitotemporal stream. Conversely, the damage to occipitotemporal regions would produce disinhibition of activity of the occipitoparietal regions and then a near/downward orienting bias.

Aim of the present study was to test the hypothesis of the involvement of the occipitotemporal stream in delayed reaching. Here we report the case of a patient, GP, who showed bilateral damage of occipitoparietal lobes. First we examined whether, according to previous studies, the occipitoparietal damage, and the relative integrity of the occipitotemporal stream, produced an attention bias toward the far space. If this was the case, we planned to examine whether the attention bias affected delayed reaching performance, shifting toward the far space remembered target locations. As a whole, these findings would add support for the involvement of the occipitotemporal stream in delayed reaching.

## Experiment 1

In the present experiment, we explored GP’s attention orienting, in the three dimensions of space, by using bisection tasks. Previous studies showed that occipitoparietal lesion could produce neglect for near/lower space (20–22). Our prediction was as follows. If GP was affected by neglect for near/lower space, she would consistently localize the subjective midpoint farther (radial lines) and more above (vertical lines) than the healthy participants.

## Material and Methods

### Case Report

**Figure 1:**
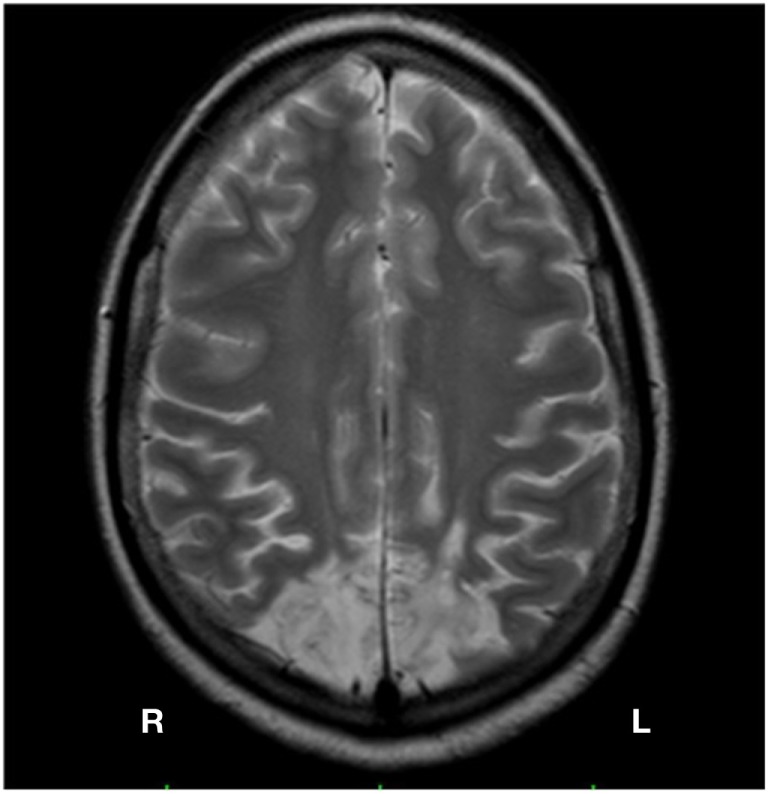
**Brain MRI of GP Bilateral lesion of the occipital cortex, slightly greater on the right side, with involvement of the adjacent parietal lobes**.

The patient, GP, is a right-handed woman with 10 years of schooling. She was 29 years old at the time of onset of first symptoms (2012), which consisted of recurrent episodes of headache. During some of the most severe headache attacks, the patient was admitted to the Emergency Unit of several Hospitals, where high blood pressure values (up to 240/120 mmHg) were always detected. She received hypotensive treatment and discharged by a few hours. In January 2013, the patient showed a new, extremely severe headache attack, followed by stupor and coma, which required admission to an Intensive Care Unit. A first brain CT scan performed few hours by admission was normal. A second CT scan, 2 days later, showed a large hypodense lesion, circumscribed by edema, involving both occipital lobes, with extension to the adjacent posterior parietal lobes. During the following days the patient showed progressive recovery of consciousness, but exhibited a severe disorder of vision resembling, at least in its first stages, the picture of a cortical blindness. Following investigations (i.e., body MRI with gadolinium) showed the presence of a left pheochromocytoma, which was surgically removed in February 2013. During the following months, the patient exhibited significant improvement of visual abilities, but showing persistent difficulties in daily living, since she referred fragmented perception of the environment, in particular when dealing with multiple stimuli and/or complex scenes, and a marked impairment in perceiving the depth. At first neurological and neuropsychological evaluation, about nine months by the onset of brain injury, the patient was alert and well oriented, with intact motility and strength of the limbs. Conversely, she referred marked disorders of visuospatial and visuomotor abilities with relevant impact on working functioning; as consequence of these difficulties, the patient was unable to start again her job of expert hairdresser. As matter of fact, she referred persistence of the fragmentary perception of the visual environment, in particular for the near space, and difficulties in catching visual targets; furthermore, these difficulties involved hand movements under visual guidance, particularly movements directed toward the lower left hemispace, or sequential movements toward objects posited at different depths. The referred symptoms led to suspect a form of a Balint (or Balint-like) syndrome. In August 2013, she underwent a brain MRI (see Figure 1) Therefore, the patient underwent clinical and neuropsychological tasks (April to June 2014) aimed to evaluate the presence of core signs of the disorder and to exclude impairment of general sensorimotor functions and disorders at cognitive domains different from visuospatial and visuomotor.

#### Visual Acuity and Visual Field

Visual acuity was quite good (right eye 8/10; left eye 9/10). At clinical evaluation the patient exhibited impairment of the visual field in both the lower quadrants, more evident on the left side; this was confirmed by a computerized campimetric examination. *Simultanagnosia*: the patient exhibited great difficulty in perceiving and naming two objects simultaneously presented. The presence of the core symptom has been established according to Wolpert’s definition and further description by Rizzo and Vecera (26), who operationally define simultanagnosia as an inability to report all the items and relation in a complex visual display, despite unrestricted head and eye movements. Following Author’s suggestion, the patient was given a picture containing a balance of information among the four quadrants (namely, the Cookie Theft). At this task, in which care was given in positioning the stimulus in the intact visual quadrants, GP correctly named single elements (e.g., the woman, the washbasin, etc.,) but she was unable to make a coherent and complete sense of pictures representing the scene. *Psychic paralysis of gaze (gaze apraxia) and optic ataxia*: they were assessed following a modified Kas et al.’s procedure (27). The patient sat at a distance of about 50 cm in front of the examiner and was asked to fix his nose. Afterward, the examiner moved a target stimulus (i.e., a coin) through the four visual quadrants. Care was given in avoiding to position the target in quadrants of the visual field with denser hemianopia. The patient was then asked to move her eyes in the direction of the coin. GP failed to move her eyes in the direction of the coin in any of the four visual quadrants, with more relevant difficulties (as clinically shown by more evident “erratic” eye movements) for the lower quadrants. This supported the diagnosis of psychic paralysis of gaze. Optic ataxia was assessed by asking the patient, whose starting position (SP) was the same of the previous task, to touch the coin with a designated hand (left or right). The examiner placed the coin one at time in each of the four visual quadrants. GP exhibited relevant difficulties in accurately reaching the target with either hands on the first attempt in all of the four visual quadrants. From the other hand, no reaching difficulties were shown with both arms if the stimulus was centrally posited and correctly foveated. These results supported diagnosis of optic ataxia. *Proprioception*: the static and dynamic proprioception of the upper limbs were assessed following methods by Blangero et al. (15), i.e., applying a slow passive movement in flexion or extension (the test included 25% catch trials) on each joint serially (index, wrist, elbow, shoulder), whilst the patient kept her eyes closed. It was asked her: (i) whether she perceived a movement; (ii) in which direction; (iii) to reproduce the single joint angles with the other limb. The patient showed no difficulties in performing the task. The patient did not show clear disorders of main cognitive domains other than visuospatial. She had a corrected score (CS) of 25.75 (cutoff: 23.8) at the Mini-Mental State Examination (28). Executive functions resulted intact as supported by normal CS = 15.28 (cutoff: 12.03) at the Frontal Assessment Battery (29, 30). Oral language was intact; in particular, the patient was able in naming all the visual stimuli (pictures representing object/animals, actions, colors) from a standard language examination battery [ENPA; (31)] so excluding an overt visual agnosia. At the same battery, although with mild slowness, she did not produce errors in reading aloud single words, non-words and sentences. The patient did not show clinical evidence of prosopagnosia. She always recognized the faces of the members of the clinical staff without the aid of their voices, if the face had been previously foveated. Verbal episodic memory showed no impairment. GP scored within the normal range at both immediate (CS = 35.1; cutoff: 28.53) and delayed recall (CS = 6.9; cutoff: 4.69) of the Rey’s 15 words list. Performances at verbal short-term memory were within the lower limits (CS digit span forward = 3.75; cutoff: 3.75). From the other hand, the patient showed significant impairment at tests of visual short-term memory (CS Corsi span = 1.75; cutoff: 3.50). GP did not show difficulties in basic visual discrimination processes since she performed in normal range at a test of scrawl comparison (CS = 23.85; cutoff: 21), nor at tasks for ideomotor apraxia; however, she resulted grossly impaired at tasks, namely constructive apraxia, requiring to accurately analyze the visuospatial characteristics of the stimulus to reproduce. This was assessed by stimuli from the Mental Deterioration Battery (32), requiring to copy designs without (CD) and with landmarks (CDL). GP had CSs of 3.7 (cutoff: 7.18) at CD task and 20.5 (cutoff: 61.85) at CDL.

### Control Group

Twelve healthy, right-handed females took part in the experiment. Their mean age was 31.1 years (range 28–36; SD 2.7). All the participants reported having normal or corrected-to-normal vision. The experiment was approved by the ethics committee of the “Azienda Ospedaliera Universitaria, Seconda Università di Napoli” and was performed in accordance with the 1964 Declaration of Helsinki (RDn 1185 of July 27, 2011). Participants gave written informed consent to take part in the study.

### Stimuli

**Figure 2:**
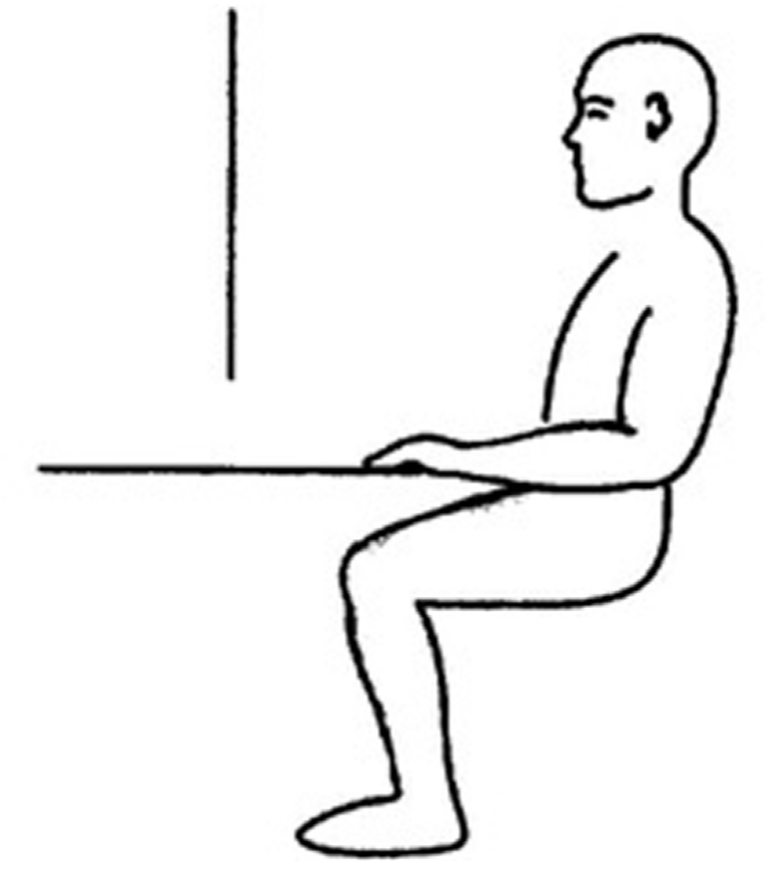
**Placement of line bisection stimuli in relation to subject**.

The stimuli were black lines, 1 mm wide, drawn and centered on white paper 29.7 cm × 21.0 cm. Lines could be of six different lengths (15, 17.5, 20, 22.5, 25, or 27.5 cm) and were presented along the three orthogonal axes: horizontal, radial, and vertical. Horizontal and radial lines were presented in the transverse plane, on the table top. Horizontal lines were oriented along the frontal plane, radial lines along the midsagittal plane. Both horizontal and radial lines were placed 35 cm below eye level and their midpoint was 30 cm from subject’s body. Vertical lines were presented on a wall, 30 cm front the subject, at the intersection of the frontal and midsagittal plane, and their midpoint was at the subject’s eye level (see Figure 2).

### Procedure

The participants sat in a comfortable chair in front of a table, where the stimuli were placed one at a time. They were asked to explore the full extent of the test line and then to localize and mark the subjective midpoint using a pencil held with their right or left hand. The task was performed by GP in two sessions, on two different days. In the first session, GP used the right hand, in the second session the left hand. Healthy participants performed the task in a single session. Half of the healthy participants used first the right hand and then the left hand; the other half vice-versa. Participants bisected a total of 72 lines [three spatial conditions (horizontal, radial, vertical) × six line lengths (15, 17.5, 20, 22.5, 25, 27.5 cm) × four presentations]. For each hand condition, the lines were grouped according to spatial orientation into three blocks. The order of the blocks so as, in each block, the order of the line lengths was randomized.

The length of the left side of the bisected line (i.e., from the left end of the line to the subject’s mark) was measured to 0.5 mm accuracy. This measurement was converted to a standardized score, the percentage deviation score, using the following formula: [(measured left half − true half)/true half] × 100. This procedure is comparable with that used in other studies (33, 34) and takes individual line length into account. This transformation yielded (+) values for marks placed to the right of (horizontal lines), farther than (radial lines), or above (vertical lines) of the true center and (−) values for marks to the left of, nearer than, or below the true center. For control group, data for each spatial condition were pooled across line lengths.

### Statistical Analysis

For each condition, GP’s performance was compared with that of the control group by means of a modified two-tailed *t*-test to small control sample size (35, 36).

Furthermore, for each spatial axis, one-sample, two-tailed *t*-tests were also performed comparing GP’s bisection errors (df = 23) with the null set (true midpoint) to investigate the direction of misbisection. Analogously, controls’ bisection errors (df = 11) were compared with the null set. Significance level was fixed at *p* < 0.008 on a Bonferroni basis after considering the number of comparisons. The effect size has been checked by the Cohen’s *d*.

## Results

**Figure 3:**
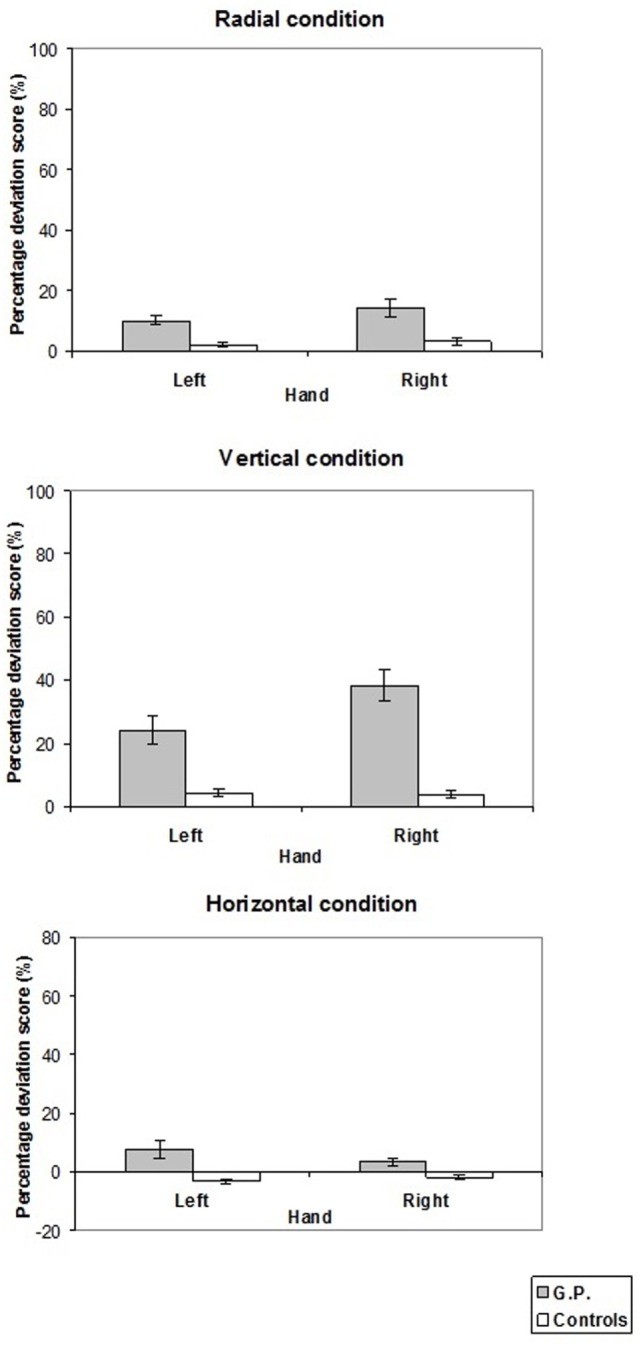
**Pattern of percentage deviation score measured in experiment 1**. On the *y*-axis, (+) values indicate that the subjective midpoint was located farther than (radial lines), above (vertical lines), to the right of (horizontal lines) the true center; (−) values to the left of, nearer than, or below the true center. Mean values are shown with SE (*bars*).

The mean values of line bisection errors of GP and the control group are shown in Figure 3.

In the radial condition, GP located the subjective midpoint farther than the control group with both the left (10.2 vs. 2.0%, *t* = 2.81, *p* < 0.02) and right (14.2 vs. 3.1%, *t* = 2.73, *p* < 0.02) hand. Analogously, in the vertical condition, GP located the subjective midpoint more above than the control group with both the left (24.1 vs. 4.2%, *t* = 4.55, *p* = 0.01) and the right (38.4 vs. 3.8%, *t* = 8.31, *p* < 0.001) hand. In the horizontal condition, when the left hand was used GP bisected lines more to the left than the controls (7.7 vs. −3.0%, *t* = 3.81, *p* < 0.005). Conversely, bisection performance did not differ between GP and controls when the right hand was used (3.5 vs. −1.9%, *t* = 1.67, NS).

The comparison of bisection error with the null set showed that both GP located the subjective midpoint farther (radial condition) and more above (vertical condition) the true center (radial condition, left hand: *t* = 7.62, *p* < 0.0001, *d* = 2.2, right hand: *t* = 4.67, *p* < 0.0002, *d* = 1.35; vertical condition, left hand: *t* = 5.29, *p* < 0.0001, *d* = 1.53; right hand: *t* = 7.90, *p* < 0.0001, *d* = 2.28). Also the controls in the vertical condition located the subjective midpoint more above the true center (vertical condition, left hand: *t* = 3.46, *p* < 0.006, *d* = 1.41, right hand: *t* = 3.29, *p* < 0.008, *d* = 1.34), whereas a trend toward significance was present for the radial condition (radial condition, left hand: *t* = 2.52, *p* < 0.03, *d* = 1.03, right hand: *t* = 2.73, *p* < 0.02, *d* = 1.12). Furthermore, as concerns horizontal lines, GP erred to the right of the true center with the right hand (right hand: *t* = 3.22, *p* < 0.005, *d* = 0.97), whereas a trend toward significance was present for the left hand (left hand: *t* = 2.72, *p* < 0.02, *d* = 0.78). The controls erred to the left of the true center with the left hand (*t* = −3.82, *p* < 0.003, *d* = −1.56).

## Discussion

The present experiment showed that GP localized the subjective midpoint of radial lines farther, and that of vertical lines more above, than the healthy controls. These observations agree with previous studies that showed the presence of attention orienting bias toward the far upper/space and near/lower space neglect in patients with occipitoparietal lesions (20–22). GP showed also left visuospatial neglect when she used left hand to bisect horizontal lines. Left neglect is one of the typical signs of Balint syndrome (37).

In experiment 2, we investigated whether in GP the attentional bias toward the far space affected the reaching of target locations, visually presented, in a delayed reaching task. Previous studies showed that patients with occipitoparietal lesion and optic ataxia improved their reaching performance when they were asked to reach remembered target positions (16–18). Milner et al. (16) hypothesized that this improvement was due to the relatively intact occipitotemporal stream. We explored whether in GP the increased attentional bias toward the far space (related to the integrity of the occipitotemporal stream) shifted forward the localization of remembered target locations.

## Experiment 2

Experiment 2 consisted of two tasks. First, GP performed an immediate reaching task. Then, both GP and healthy controls performed a delayed reaching task. They were asked to remember a target location, visually presented, within their reaching space. After a delay, participants were asked to reach it. We explored whether the increase of the attentional bias toward the far space detected in GP affected memory encoding of remembered target locations. If this was the case: (i) GP would make overshoot errors in delayed but not in immediate reaching task and (ii) GP, but not the control group, would make overshoot errors in delayed reaching task.

## Material and Methods

### Control Group

Eight healthy subjects who took part in the experiment 1 participated to the experiment 2. Their mean age was 30.6 years (range 28–36, SD 2.9). The experiment was approved by the ethics committee of the “Azienda Ospedaliera Universitaria, Seconda Università di Napoli” and was performed in accordance with the 1964 Declaration of Helsinki (RDn 1185 of July 27, 2011). Participants gave written informed consent to take part in the study.

### Apparatus

**Figure 4:**
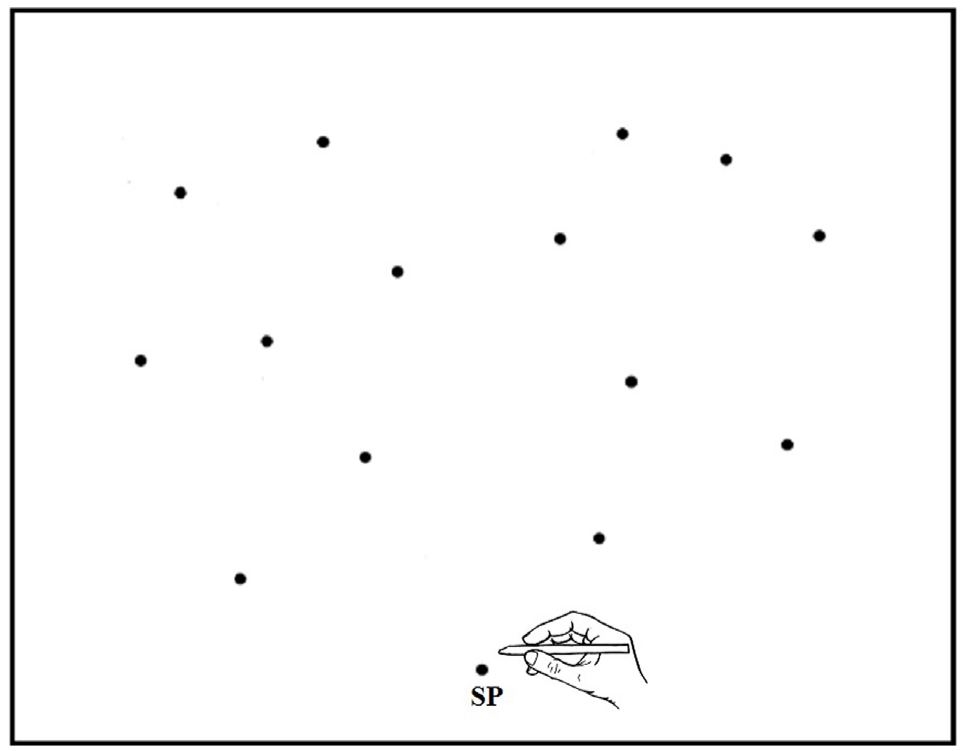
**Schematic representation of the starting position (SP) and target locations in experiment 2 (and experiment 3)**. Note that the proportions of drawing elements are not respected.

GP and healthy controls sat in a comfortable chair in front of a table on which a digitizing tablet was placed. The tablet measured 570 mm (width) × 430 mm (depth) and had an active surface of 458 × 305 mm. It was contacted with a non-inking electronic stylus. When in contact with the active surface, the position of the stylus tip was sampled at a rate of 50 Hz. Data were recorded in *X* (horizontal) and *Y* (vertical) co-ordinates with a measuring accuracy of 0.25 mm. The tablet surface was covered with a thin white card, on which the SP was drawn in black ink (a 3 mm diameter spot) at 15 cm from the trunk along the median sagittal axis. The target stimulus consisted of a flat, black disk, 5 mm in diameter. The distance and direction of each target location is shown in Figure 4. Target distance and direction were paired pseudorandomly in order to minimize possible symmetric patterns, which might assist location memory.

### Procedure

#### Immediate Reaching Task

At the beginning of each trial, GP held the stylus with her right (left) hand and placed tip of the stylus on the SP. The left (right) hand rested on her lap throughout the experiment. Then, GP closed her eyes and the experimenter placed the target stimulus on the tablet. GP opened her eyes, looked at the target, and reached it under visual control, at a natural velocity. Note that GP was free to move her eyes and head. The testing was conducted in two sessions, on two different days. In the first session, GP used the right hand, in the second session the left hand. In each session, there were 28 trials (2 hemispaces × 7 locations × 2 presentations). Target locations were randomized.

#### Delayed Reaching Task

At the beginning of each trial, both GP and healthy controls held the stylus with her right (or left hand) and closed her eyes. The left (right) hand rested on her lap throughout the experiment. The experimenter placed the stimulus on the tablet surface and the participant’s hand on the SP. The participant opened her eyes, looked at the target stimulus for 2 s, and then closed her eyes. When the 2 s retention interval had elapsed, the experimenter verbally gave the order to go. The participant was instructed to move the tip of the stylus on the tablet at a natural speed and to stop with the stylus in the remembered target position, still with eyes closed. At the end of the participant’s movement, the experimenter brought the participant’s hand back to the SP. The experimenter ensured that participants never saw the end point of their movement. The participants were free to move their eyes and head.

The task was performed by GP in two sessions, on two different days. In the first session, GP used the right hand, in the second session the left hand. In each session, there were 28 trials (2 hemispaces × 7 locations × 2 presentations). Control participants performed the task in a single session. There were two blocks of 28 trials (2 hemispaces × 7 locations × 2 presentations). Half of the participants used the right hand in the first block and the left hand in the second; the other half vice-versa. Target locations were randomized.

In both immediate and delayed reaching tasks, the dependent variables employed were: constant percentage radial (i.e., *distance*) and angular (i.e., *direction*) errors. Constant errors were measured with reference to the finger SP. For constant distance errors, reaching errors farther than the target location were assigned (+) values, whereas errors nearer than the target location were assigned (−) values. For constant direction errors, reaching errors in the direction away from the median sagittal axis, both in the left and in the right hemispace, were given (+) values. In the delayed reaching task, we measured also variable distance and direction errors. Variable distance and direction errors corresponded to the standard deviation of the constant distance and direction errors. They are an index of stability quantifying the scatter of errors and are sensitive to variability or inconsistency in reaching.

### Statistical Analysis

In immediate reaching task, to examine whether there was a consistent bias in distance and direction errors, constant errors of GP were compared with the null set, using one-sample, two-tailed *t*-tests (df = 27). Significance level was fixed at *p* < 0.0125 on a Bonferroni basis after considering the number of comparisons. The effect size has been checked by the Cohen’s *d*.

In delayed reaching task, to compare the performance of GP with that of healthy controls constant and variable errors were confronted, using a modified two-tailed *t*-test to small control sample size (35, 36).

Furthermore, constant distance and direction errors of GP (df = 27) and those of healthy controls (df = 7) were compared with the null set, using one-sample, two-tailed *t*-tests. Significance level was fixed at *p* < 0.0125 (Bonferroni correction) and the Cohen’s *d* was calculated.

## Results

### Immediate Reaching Task

The comparison of distance and direction errors with null set showed that GP reached accurately target locations in all experimental conditions (constant distance error, left hand/left hemispace: −0.91% (s.d. = 3.77%), *t* = −1.28, *p* ns; left hand/right hemispace: −0.14% (1.89%), *t* = −0.40, *p* ns; right hand/left hemispace: −0.20% (2.47%), *t* = −0.43, *p* ns; right hand/right hemispace: −0.21% (1.88%), *t* = −0.60, *p* ns; constant direction error, left hand/left hemispace: 0.09° (1.45°), *t* = 0.32, *p* ns; left hand/right hemispace: 0.39° (1.37°), *t* = 1.51, *p* ns; right hand/left hemispace: *t* = 0.46° (1.33°), *t* = 1.81, *p* ns; right hand/right hemispace: −0.43° (1.29°), *t* = −1.76, *p* ns).

### Delayed Reaching Task

**Figure 5:**
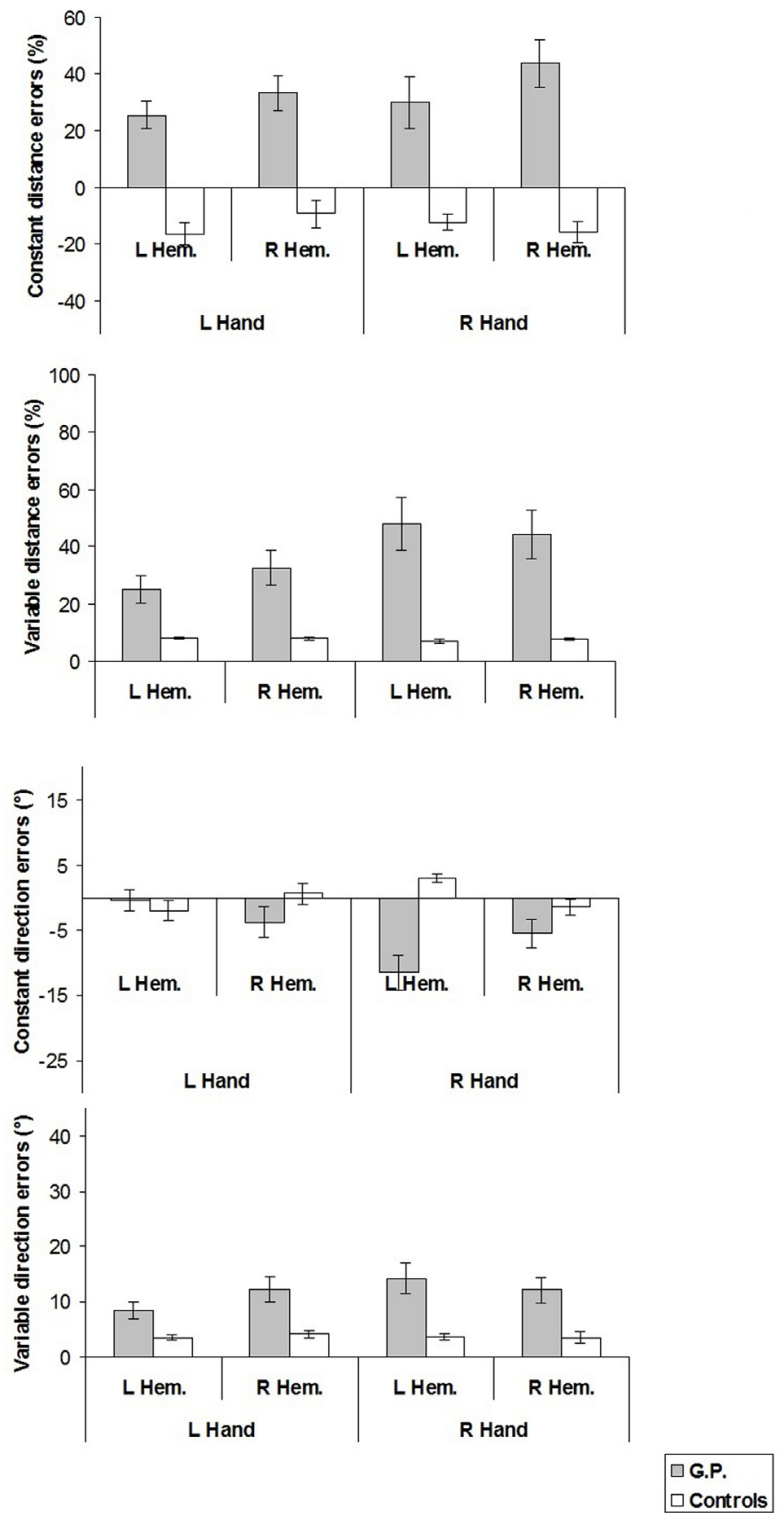
**Constant and variable distance and direction errors measured in experiment 2**. For constant distance errors, on the *y*-axis, (+) values indicate reaching errors farther than the target location, (−) values nearer than the target location, with reference to the finger starting position. For constant direction errors, (+) values indicate reaching errors in the direction away from the median sagittal axis. Mean values are shown with SE (*bars*). L, left; R, right; Hem., hemispace.

The mean values of the constant and variable distance and direction errors are shown in Figure 5.

#### Constant Distance Errors

In all experimental conditions, constant distance errors of GP differed from those of healthy participants (left hand/left hemispace, GP 25.5% vs. controls −16.5%, *t* = 3.44, *p* < 0.02; left hand/right hemispace, 33.4 vs. −9.1%, *t* = 2.95, *p* < 0.03; right hand/left hemispace, 29.9 vs. −12.4%, *t* = 5.12, *p* = 0.001; right hand/right hemispace, 43.7 vs. −16.0%, *t* = 5.26, *p* = 0.001).

Furthermore, when constant distance errors were compared with the null set, it turned out that GP significantly overshot target positions in all the conditions (left hand/left hemispace: *t* = 5.37, *p* < 0.0001, *d* = 1.43; left hand/right hemispace: *t* = 5.44, *p* < 0.0001, *d* = 1.45; right hand/left hemispace: *t* = 3.30, *p* < 0.003, *d* = 0.88; right hand/right hemispace: *t* = 5.22, *p* < 0.0001, *d* = 1.39), whereas healthy participants significantly undershot target locations in all the conditions except in the left hand/right hemispace condition (left hand/left hemispace: *t* = −4.06, *p* < 0.005, *d* = −2.03; left hand/right hemispace: *t* = −1.92, ns; right hand/left hemispace: *t* = −4.53, *p* < 0.003, *d* = −2.25; right hand/right hemispace: *t* = −4.24, *p* < 0.004, *d* = −2.11).

#### Variable Distance Errors

In all experimental conditions, variable distance errors of GP were significantly greater than those of healthy participants (left hand/left hemispace: GP 25.2% vs. Controls 8.3%, *t* = 11.38, *p* < 0.001; left hand/right hemispace: 32.5 vs. 8.1%, *t* = 14.38, *p* < 0.001; right hand/left hemispace: 48.0 vs. 7.0%, *t* = 14.87, *p* < 0.001; right hand/right hemispace: 44.4 vs. 7.7%, *t* = 28.83, *p* < 0.001).

#### Constant Direction Errors

Only in the right hand/left hemispace condition, constant direction error differed between GP and healthy participants (GP −11.5° vs. Controls 3.0°, *t* = 4.45, *p* < 0.01).

From comparisons of constant direction errors with the null set, it appeared that in right hand/left hemispace condition GP deviated significantly toward the midsagittal axis (*t* = −4.27, *p* < 0.0003, *d* = −1.14), whereas healthy participants deviated significantly away the midsagittal axis (*t* = 4.75, *p* < 0.003, *d* = 2.37).

#### Variable Direction Errors

In all experimental conditions, variable direction errors of GP were significantly greater than those of healthy participants (left hand/left hemispace, GP 8.5° vs. Controls 3.4°, *t* = 4.01, *p* = 0.005; left hand/right hemispace, 12.3° vs. 4.2°, *t* = 4.77, *p* = 0.002; right hand/left hemispace, 14.3° vs. 3.6°, *t* = 7.21, *p* < 0.001; right hand/right hemispace, 12.1° vs. 3.5°, *t* = 4.27, *p* < 0.005).

## Discussion

In immediate reaching task, GP reached accurately target locations presented in the left or right hemispace, with her right or left hand. Note that GP could foveate target locations. This result agreed with its neurological examination: she misreached with her right or left hand stimuli presented in peripheral vision, whereas reached accurately stimuli presented in foveal vision. Previous researches reported that pointing in central vision is essentially unimpaired in optic ataxia (13, 14).

In delayed reaching task, GP and the healthy participants exhibited an opposite patterns of errors. Whereas GP overshot remembered target locations, the healthy participants undershot them. Taken together, the results of both immediate and delayed reaching tasks suggest that in GP the attentional bias toward the far space, detected in experiment 1, shifted forward remembered target locations, visually specified. However, it is important to note that in delayed reaching task, while the target was visually presented, the movement occurred without vision control. In this case, movement execution was monitored using proprioceptive information. Then, it is possible that the use of proprioceptive information contributed, or produced, overshoot errors. To examine whether this was the case, we performed the experiment 3.

## Experiment 3

In this experiment, both GP and healthy participants were asked to remember target locations proprioceptively presented. The experimenter passively moved the hand of blindfolded participants up to a target location. After a delay, participants were required to reach the remembered target location. Our prediction was as follows. If GP overshot target locations, this would suggest that the overshoot errors observed in experiment 2 might depend on the use of proprioceptive information. Conversely, if GP did not overshoot target locations, this would further support the hypothesis that the overshoot errors observed in experiment 2 were related to memory encoding of visual target locations.

## Material and Methods

### Control Group

The same healthy subjects who had taken part in the experiment 2 participated in the experiment 3. The experiment was approved by the ethics committee of the “Azienda Ospedaliera Universitaria, Seconda Università di Napoli” and was performed in accordance with the 1964 Declaration of Helsinki (integration with Decree 922 October 22, 2013 of RDn 1185, July 27, 2011). Participants gave written informed consent to take part in the study.

### Apparatus

The apparatus was the same as experiment 2.

### Procedure

GP and healthy controls were blindfolded during the whole experimental session. They held the stylus with the right (or left) hand. The left (right) hand rested on her lap throughout the experiment. The experimenter placed the participants’ hand on the SP and passively moved it up to the end position (criterion movement, CM), and again to the SP. After 2 s, participants were required to reach actively the remembered CM end-position, at a natural velocity. The CM-end positions corresponded to the target locations of experiment 2.

The task was performed by GP in two sessions, on two different days. In the first session, GP used the right hand, in the second session the left hand. In each session, there were 28 trials (2 hemispaces × 7 locations × 2 presentations). Control participants performed the task in a single session. There were two blocks of 28 trials (2 hemispaces × 7 locations × 2 presentations). Half of the participants used the right hand in the first block and the left hand in the second; the other half vice versa. CM end point locations were randomized.

The dependent variables employed were: constant percentage radial (i.e., *distance*) and angular (i.e., *direction*) errors. Constant errors were measured with reference to the finger SP. For constant distance errors, reaching errors farther than the CM end point (overshoot errors) were assigned (+) values, whereas errors nearer than the CM end point (undershoot errors) were assigned (−) values. For constant direction errors, reaching errors in the direction away from the median sagittal axis, both in the left and in the right hemispace, were given (+) values.

### Statistical Analysis

To compare the performance of GP with that of healthy controls constant and variable errors were confronted, using a modified two-tailed *t*-test to small control sample size (35, 36).

Furthermore, constant distance and direction errors of GP (df = 27) and healthy controls (df = 7) were compared with the null set, using one-sample, two-tailed *t*-tests. Significance level was fixed at *p* < 0.0125 (Bonferroni correction), and the effect size has been checked by the Cohen’s *d*.

## Results

**Figure 6:**
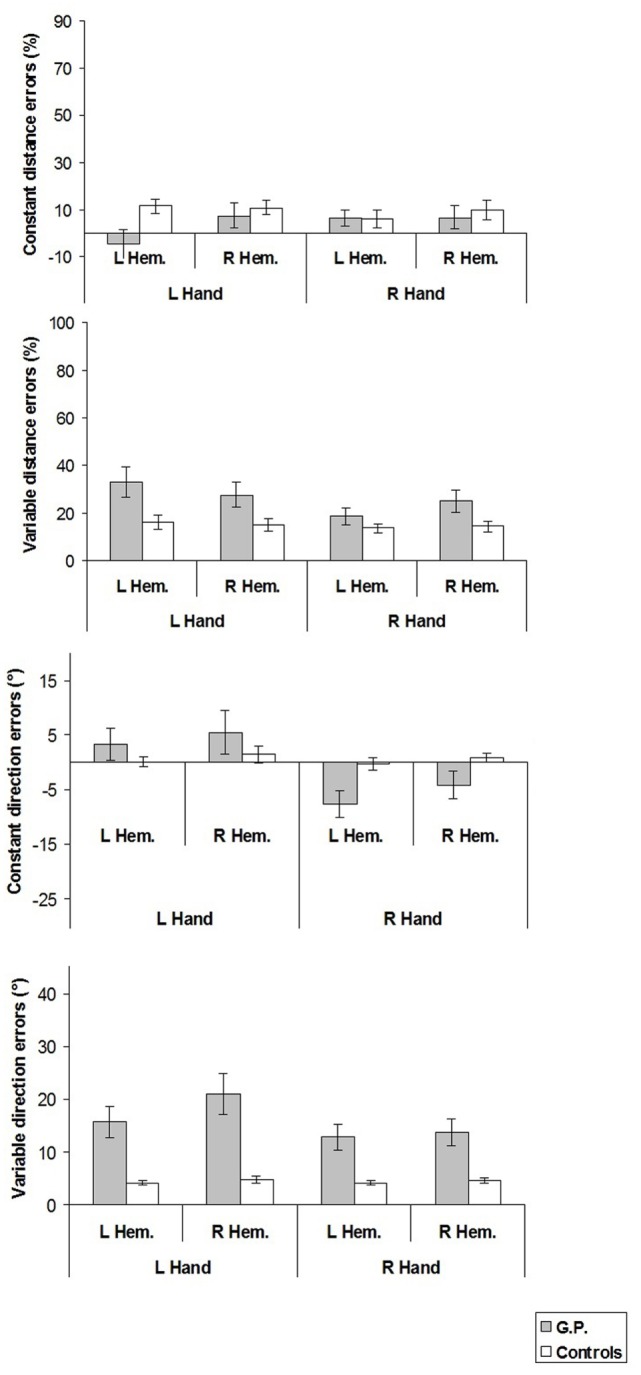
**Constant and variable distance and direction errors measured in experiment 3**. For constant distance errors, on the *y*-axis, (+) values indicate reaching errors farther than the criterion movement end point, (−) values nearer than the criterion movement end point, with reference to the finger starting position. For constant direction errors, (+) values indicate reaching errors in the direction away from the median sagittal axis. Mean values are shown with SE (*bars*). L, left; R, right; Hem., hemispace.

The mean values of the constant and variable distance and direction errors are shown in Figure 6.

### Constant and Variable Distance Errors

Constant and variable distance errors did not differ between GP and healthy controls.

When the constant distance error was compared with the null set, it was found that healthy controls overshot target location with left hand in both hemispaces (left hand/left hemispace: *t* = 3.69, *p* < 0.008, *d* = 1.85; left hand/right hemispace: *t* = 3.59, *p* < 0.009, *d* = 1.80).

### Constant Direction Errors

Constant direction errors of GP did not differ from those of healthy controls.

From the comparison of constant direction errors with the null set, it was found that GP significantly deviated toward the midsagittal axis in the right hand/left hemispace condition (*t* = −3.19, *p* < 0.004, *d* = −0.85).

### Variable Direction Errors

In all experimental conditions, variable direction errors of GP were significantly greater than those of healthy participants (left hand/left hemispace, GP 15.6° vs. Controls 4.0°, *t* = 9.94, *p* < 0.001; left hand/right hemispace, 20.9° vs. 4.7°, *t* = 8.04, *p* < 0.001; right hand/left hemispace, 12.9° vs. 4.1°, *t* = 6.91, *p* < 0.001; right hand/right hemispace, 13.7° vs. 4.6°, *t* = 5.72, *p* = 0.001).

## Discussion

Both constant distance and direction errors of GP did not differ from those of the healthy participants. Furthermore, when distance errors were compared with null set it turned out that GP reached accurately target locations. These observations suggested that the ability in extracting CM proprioceptive information, and the use of proprioceptive feedback to monitor target-reaching, was relatively preserved. Thus, the results of the present experiment support the hypothesis that the overshoot errors observed in experiment 2 were related to memory encoding of visual target locations, rather than to an impairment in processing of proprioceptive information.

## General Discussion

The main findings of the present study were the following: (i) GP misbisected radial lines farther than, and vertical lines above, the true center (experiment 1); (ii) she overshot remembered target locations, visually presented (experiment 2); (iii) GP reached accurately remembered target locations, proprioceptively presented (experiment 3).

In experiment 1, GP and healthy controls bisected lines oriented in the three dimensions of space. Both GP and the control group bisected radial lines farther than, and vertical lines above the true center. However, the bisection errors were greater in GP than in the controls. The presence of a directional bias toward far/upper space in the control group is in line with previous studies (23, 38–40). Shelton et al. (23) attributed this bias to perceptual/attentional factors. During visual exploration, attention is preferentially distributed away from the body (“far peripersonal space”), since the visual system is tuned to detect distant stimuli (23). Another factor that could be involved in producing the bisection bias lies in the nature of the task itself. Line bisection task is a perceptual-motor task. It consists of several stages that may be intuitively summarized as follows: (1) visual scanning of the line and localization of the subjective midpoint, (2) planning and execution of the movement directed to mark the subjective midpoint. It was suggested that the localization of the subjective midpoint would require an allocentric estimation (41–45). In line bisection, the eyes tend to fixate near the center of the line during the majority of time (46, 47). In this way, the line is divided into two segments whose magnitude is compared (48). Comparing two objects is assumed to be an allocentric task mediated by the occipitotemporal system (41). Then, it is possible that the selective involvement of the occipitotemporal system in localizing the subjective midpoint shifted the latter forward/upward. However, the shifting of the subjective midpoint was greater in GP than in healthy controls. This was likely due to an unbalance of attention orienting along the near/far and lower/upper dimensions of space caused by the occipitoparietal lesion and the relative integrity of occipitotemporal areas. The occipitoparietal lesion would produce a deficit in attention orienting toward the near/lower space (20–22). Furthermore, the lack of inhibition by occipitoparietal system of occipitotemporal system might contribute to increase the forward/upward attention bias (25). Then, it is plausible that the bilateral occipitoparietal lesion in GP led to an over-reliance on occipitotemporal stream, determining an increase of the forward/upward attentional bias.

In experiment 2, we examined whether such attentional bias shifted forward remembered visual target locations, being occipitotemporal areas also involved in memory encoding of target location. According to our hypothesis, the results showed that GP overshot remembered target locations. Conversely, healthy controls showed undershoot errors, in line with previous studies (49–52). Apparently, the performance of GP seemed to disagree with that previously observed in other patients with optic ataxia (16–18, 53). These patients showed an improvement in reaching when a delay was imposed (17, 18, 53). Conversely, GP accurately reached target locations in immediate condition, but overestimated remembered target locations. The improvement in delayed reaching task of patients with optic ataxia was first reported by Milner et al. (16) who described the case study of AT. The patient presented bilateral parietal damage extending to the occipital lobes and severe optic ataxia for targets in her peripheral visual field (16, 53). Milner et al. (16, 53) observed that AT made large errors when she pointed to targets immediately upon their presentation, but her performance improved when she was required to delay a few seconds before responding to remembered target location. A similar improvement was observed in few other patients with optic ataxia due to unilateral or bilateral lesions of the occipitoparietal cortex, namely, IG (53) OK (17), GH and US (18). Milner et al. (16, 53) suggested that the improvement of the pointing performance in the delayed conditions was due to the sparing of occipitotemporal areas, which partially compensated parietal damage, by retaining information about target location. Some factors could be intervened in causing the discordance between our findings and those reported previously in optic ataxia. First, GP was affected not only by optical ataxia, but also by neglect for near space. Conversely, the clinical descriptions of the above case studies did not report the presence of neglect along the near/far axis (16–18, 53). Then, the overshot errors observed in GP might depend on the presence of neglect for near space and attentional bias toward the far space. Second, in immediate condition, GP could foveate the target while performing the reaching movement. The above case studies performed the reaching movements while fixating a LED (16–18, 53). Their pointing responses were directed accurately when made to the fixation point and for targets close to the fixation point, but became progressively less accurate with increasing eccentricity. These observations are in line with previous studies that showed that pointing in central vision is known to be essentially unimpaired in optic ataxia (13, 14). The results of experiment 2 also suggested that visuomotor integration in GP was less efficient than in controls. This was supported by the observation that in all hand/hemispace conditions both distance and direction variable errors were greater in GP than in healthy controls.

It is important to note that in delayed visual reaching task, GP reached the remembered target location with her eyes closed. In this case, she used proprioceptive feedback to monitor spatial displacement of arm while reached the target. Proprioception provides the basis for the conscious perception of limb position and velocity when the eyes are closed (54–58). In a previous study, Blangero et al. (15) showed that proprioceptivo-motor integration was impaired in two patients, OK and CAN, with unilateral parietal posterior cortex damage and optic ataxia. Patients had a severe deficit in reaching for proprioceptive targets and in extracting proprioceptive information about the spatial location of the ataxic hand. Then, it was possible that in experiment 2 an impairment in monitoring proprioceptively target-reaching could be responsible for overshoot errors observed in GP. We studied the ability of GP in reaching remembered target locations, proprioceptively specified. The results showed that GP reached accurately remembered proprioceptive target locations, supporting the view that the forward attentional bias was restricted to visual modality. However, in all hand/hemispace conditions direction variable errors were greater in GP than in healthy controls. This observation suggested that proprioceptivo-motor integration in GP was less efficient than in controls.

The present study is not free from some criticism. The main limitation is that it is a single case report. From the other hand, we would underline that the syndrome observed in our patient is relatively rare to observe. Further studies, possibly on homogeneous small groups of patients with optic ataxia, will better address the role of the ventral stream in memory for spatial locations.

In conclusion, the present study suggests that the occipitoparietal damage, and the relatively intact occipitotemporal region, may produce an attentional orienting bias toward the far/upper space that shifts selectively, in the same direction, remembered visual target locations to be reached. As a whole, these findings further support the view of an involvement of the occipitotemporal stream in delayed reaching. Furthermore, the observation that in both delayed reaching tasks the variable errors were greater in GP than in the controls suggests that it is possible to detect in optic ataxia not only a visuo- but also a proprioceptivo-motor integration deficit.

## Ethics Statement

The experiment was approved by the ethics committee and was performed in accordance with the 1964 Declaration of Helsinki. Participants gave written informed consent to take part in the study.

## Author Contributions

SC, GM, MM, and AI: conceived the study, participated in its design and wrote the manuscript. FA, EG, and FR: contributed to the conception and design. IV, AM, and VM: drafted the article and revised it critically for important intellectual content. SC and GM: final approval of the version to be published. All authors read and approved the final manuscript.

## Conflict of Interest Statement

The authors declare that the research was conducted in the absence of any commercial or financial relationships that could be construed as a potential conflict of interest.
